# Phase behavior study on gas hydrates formation in gas dominant multiphase pipelines with crude oil and high CO_2_ mixed gas

**DOI:** 10.1038/s41598-020-71509-6

**Published:** 2020-09-08

**Authors:** Jai Krishna Sahith Sayani, Srinivasa Rao Pedapati, Bhajan Lal

**Affiliations:** 1 Department of Mechanical Engineering, Universiti Teknologi PETRONAS, 32610 Bandar Seri Iskandar, Perak Malaysia; 2 CO2 Research Centre (CO2RES), Universiti Teknologi PETRONAS, 32610 Bandar Seri Iskandar, Perak Malaysia; 3 Department of Chemical Engineering, Universiti Teknologi PETRONAS, 32610 Bandar Seri Iskandar, Perak Malaysia

**Keywords:** Energy science and technology, Engineering

## Abstract

This research is focused on understanding the phase behavior of gas hydrate formation in the gas dominant multiphase pipelines containing mixed gas with high CO_2_, crude oil, and deionized water. The experimental conditions are in the pressure range of 3–7 MPa with water cut as 20% of the volume. Initially, the effect of high CO_2_ content in natural gas on the phase boundary conditions of hydrates is studied through simulation (CSMGEM software) and experiments. Later, an additional phase of crude oil was introduced, with 15% of the volume to study the multiphase system. From the experimental analysis, thermodynamic equilibrium conditions were found, and the hydrate-liquid–vapor-equilibrium (HLVE) curves were drawn. The phase behavior is comprehended by comparing the HLVE curves of pure and multiphase systems. It is found that the high CO_2_ content tends to promote the gas hydrate formation. Based on the results, temperature variance and enthalpy of formation were calculated for the multiphase system. With a difference of 1.32 average temperature variance, the multiphase system exhibits inhibition. A basic statistical regression model was made to predict the gas hydrate formation in multiphase transmission pipelines. This work helps in understanding the effect of a new phase on gas hydrate formation.

## Introduction

Gas hydrates are ice-like solid compounds in which gas molecules are sheathed in cages and are formed by hydrogen-bonded water (H_2_O) molecules and stabilized by van der Waals forces^1,2^. Usually, at high pressure and low-temperature conditions, these non-stoichiometric compounds are developed. Methane (CH_4_), carbon dioxide (CO_2_), ethane (C_2_H_6_), propane (C_3_H_8_), hydrogen sulfide (H_2_S), and butane (i-C_4_H_10_) are among the typical hydrate formers frequently encountered in deep-sea situations. Throughout the extraction of oil and gas, water is often present together with an abundance of hydrocarbons in proximity. Thus, clathrate hydrates are formed within the oil, gas, and multiphase flow lines mostly under thermodynamically favorable conditions (low temperature and high pressure) such as the deep-sea conditions^3–5^.

One of the significant problems in flow assurance is the formation of gas hydrates. Gas hydrate formation leads to the blockage in pipelines, therefore becoming the reason for the loss in hydrocarbon production, transportation, and processing facilities. Flow assurance challenges become more significant as oil and gas explorations field development has progressed into deeper water (> 500 m), where longer pipelines in hostile operating environments are prone to gas hydrate formation. Multiphase flow through pipelines contends with many engineering applications besides installations^6^. In petroleum production and processing, chemical processing, problems associated with the concurrent flow of multiple phases through flowlines has been a long-time interest^7^. This interest has risen substantially in recent years due to solicitations to new developments in petroleum production and refining. By transporting multiple phases like gas, oil, and water together from wells in satellite fields to existing processing facilities, it would be more economical for expanding production. The hydrate formation in transmission pipelines, which leads to blockage, is always the main issue affecting transmission safety. During production, processing, and gas transmission, there is a high possibility for the plugging of pipelines due to hydrate formation, which poses the major flow assurance challenge^8^.

Many conventional hydrate mitigation methods are adapted over the years^9–12^. However, many of them are either incompetent or required an enormous amount of chemical solvents occasioning in high operational cost along with the severe environmental impact on operating gas and oil facilities^13^. Besides, the existing inhibitors are still not able to provide an economical solution particularly at high pressure and rapid subcooling conditions. Also, there is no detailed research regarding multiphase systems, which are mostly operating conditions during natural gas production. Likewise, none of the previous investigations dealt with the hydrate phase behavior modeling of the multiphase mixed gases system. Hydrate phase behavior modeling is required to optimize the usage of chemical inhibitors.

Currently, there are several research conclusions available on the formation of gas hydrates and their degree of plugging in natural gas pipelines. However, the research related to them in multiphase pipelines or multiphase flow is very minutely discussed^14,15^. Meantime, adjudicating the formation of gas hydrates and the determination of the formation region is essential. The phase behavior study is so important as it helps in the development of dynamic prediction capabilities with the existing simulation tools. Also, quantification of the degree of blockage in pipelines due to gas hydrate formation plays a significant role for safer transmission in pipelines^16^.

The initial discussion about the gas hydrates formation in multiphase flow lines was noted to be in the 1980s. As a part of the Conoco Hydrate program in Colorado School of Mines, a theoretical study has been done on gas hydrates formation in the multiphase system. From this, phase behavior of hydrate formation is studied. The temperature and pressure conditions, the quantity of liquid in a gas pipeline to form hydrates were estimated. Later, the study of multiphase pipelines and gas hydrate formation in them drawn significant interest. The research was carried out and found that the flow parameters like velocity and discharge of fuel in the pipelines also affect the kinetics of gas hydrates. Various flow velocities ranging from 0 to 5 m/s is considered for experimental evaluation for gas hydrate formation. It was found that the higher the velocity, the faster the gas hydrate plugging in the pipeline^17^.

In multiphase flow, the effect of the various flow regimes like bubble flow, annular flow and slug flow are investigated to predict the growth kinetics of gas hydrates^18^. With the advancements in software simulation capabilities, the prediction of gas hydrates with theoretical modelling increased vividly. Also, the application of computational fluid dynamics (CFD) increased and a prediction model is proposed to evaluate the particle deposition on the pipeline walls during gas hydrates formation^19^. The development and proposal of hydrodynamic models to predict the gas hydrates formation in multiphase pipelines eased the path for advanced research studies^20^. There is an upsurge in the experimental investigation and analysis as most of the numerical modelling as well as simulations by commercial software’s are to be validated by experimental data or real time field data^21^.

Studies increased about the gas hydrate formation/dissociation in multiphase systems as the experiments were carried out with fuel oils like Diesel. Various researchers also carried out work on emulsion pipelines with different water cuts to experimentally find the phase behavior conditions of gas hydrates in multiphase pipelines. The mixed gas system with CH_4_, C_2_H_6_, and C_3_H_8_ is used, and the formation of gas hydrates is studied in the crude oil emulsion system with 50–80% water cuts variations. It has been found that at 50% water cut, the formation of gas hydrates was high when compared to that of 80% water cut^22^. The research about the occurrence of the methane hydrate in dispersed oil medium by experimental and simulation has drawn attention furthermore. Research on gas hydrate formation in multiphase pipelines with gas dominant or oil dominant system containing black oil or crude oil is later done vividly to estimate the real-time subsea conditions and the kinetics of gas hydrates^23^. So, these days, experiments and simulation on the gas dominant multiphase pipelines with the presence of crude oil and different water cuts is the trend of research^24^. Currently, due to the decrease in the quality of the natural gas wells, the quality of natural gas is depleted, and the presence of high carbon dioxide is observed^25^.

In this work, the phase behavior of gas hydrate formation in the gas dominant multiphase pipelines containing mixed gas with high CO_2_, crude oil, and deionized water has been studied under the pressure range of 3–7 MPa with water cut as 20% of the volume through the means of experiments, simulation, and a basic statistical regression model. The mixed gas studied consists of 70% carbon dioxide, 26% methane, 2% nitrogen, 1% propane, and 1% butane.

## Discussion and results

### Verification of experimental conditions

**Figure 1:**
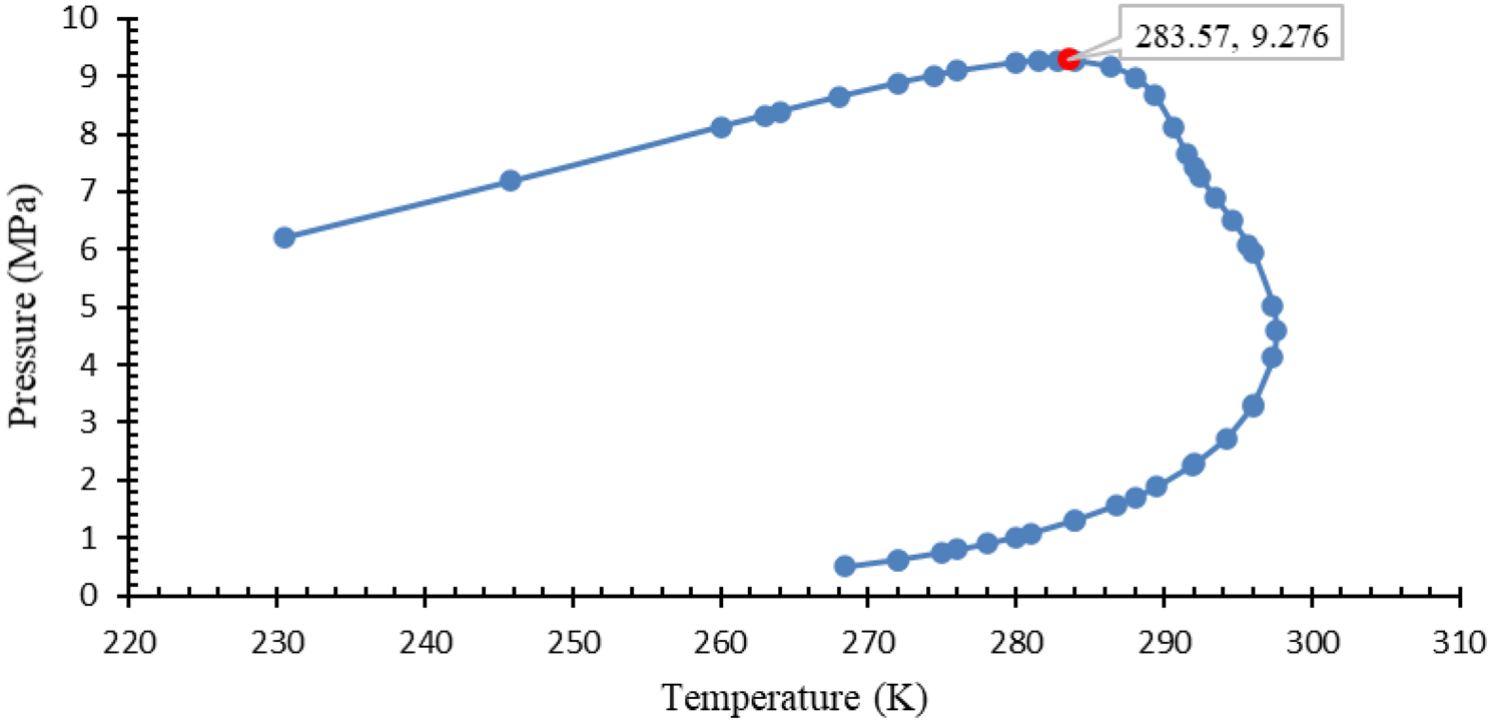
Phase diagram of natural gas with 70% CO_2_ content.

Generally, natural gas pipelines are operated at extremely high pressures during their transmission. They are operated around 1–10 MPa depending upon the composition of the natural gas as containing heavier carbon compound gases impacts the pipeline flow conditions^26^. But, when there is a high carbon dioxide content in natural gas, these pipelines cannot be operated at same environments due to acidic nature of carbon dioxide gas and low saturation point of its gaseous state. The gas once liquified impacts the pipelines due to corrosion and other issues. So, to understand the operating conditions of high CO_2_ content natural gas pipelines, a phase diagram has been constructed for natural gas with high CO_2_ using the PVTSIM software package. This helps in understanding the critical point of the gas used for this analysis and helps in validating the experimental conditions chosen. The phase diagram is presented in Fig. 1.

From the phase diagram, the critical point for the gas is found to be 283.57 K and 9.276 MPa. So, from this, it can be concluded that the hydrate region of the chosen gas is below the curve towards the axis. From this, it can be verified that the experimental conditions to replicate the real-time operating conditions of natural gas with high CO_2_ are acceptable.

### Validation of experimental setup

**Figure 2:**
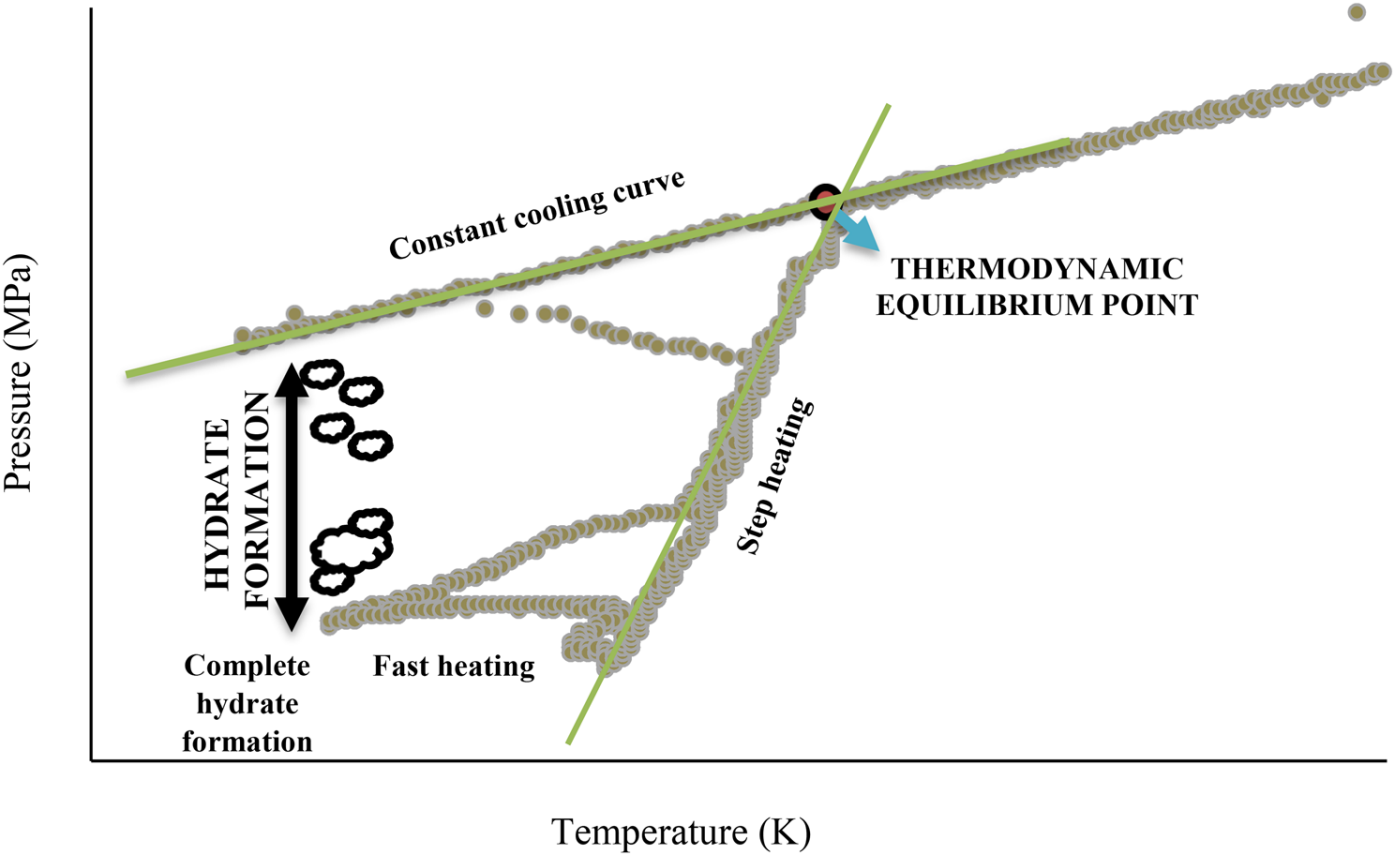
Typical pressure—temperature profile measured during the experiment (T-cycle).

**Figure 3:**
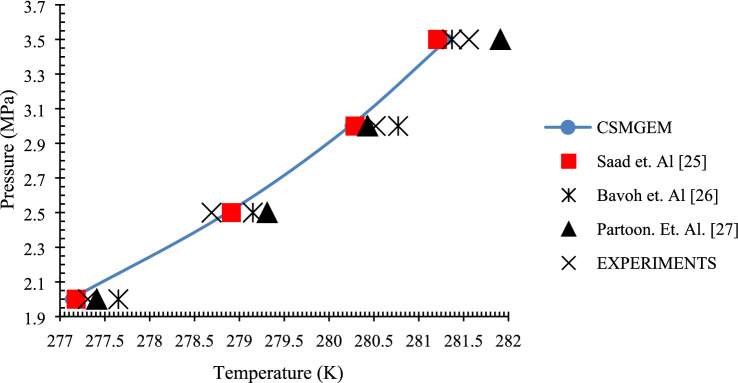
Validation of equipment with carbon dioxide gas.

**Figure 4:**
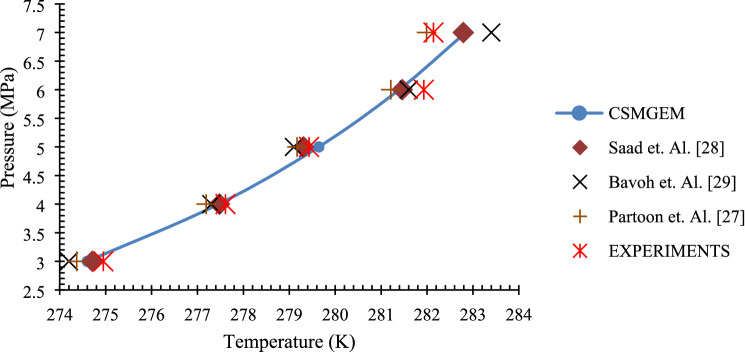
Validation of equipment with methane gas.

Thermodynamic equilibrium conditions are analyzed from the plot of T-cycle curves. The cyclic plotting of the pressure and temperature changes in the reactor brings up the T-cycle. The cooling, stabilization, and heating due to which the pressure and temperature fluctuations were recorded. Typically, the thermodynamic equilibrium point is usually the temperature and pressure condition where the heating and cooling curve meet^27^. This T-cycle curve is represented in Fig. 2. Experiments are conducted with carbon dioxide gas and methane gas at various pressure conditions to obtain the thermodynamic equilibrium points. From these points, the HLVE plot is drawn and compared with the simulation results of CSMGEM and literature. The results for the carbon dioxide gas and methane gas are presented in Figs. 3 and 4, respectively. For both carbon dioxide and methane gas, the equipment gave results that are in line with the CSMGEM results as well as literature results. The literature results for validation when carbon dioxide gas is used were taken from^28–30^, whereas for methane gas, the results are made from^30–32^. The simulation values of nitrogen and butane are not mentioned as the considered pressure range is out of their respective hydrate zones^33^. According to the literature, the minimum pressure required for nitrogen to maintain at the hydrate zone is 10.5 MPa. For butane, the value of pressure is out of the range of the considered pressure. When the thermodynamic equilibrium points are defined by simulation, the HLVE curve has been plotted.

### Phase behavior based on experiments

#### Effect of high CO_2_ in natural gas system

**Figure 5:**
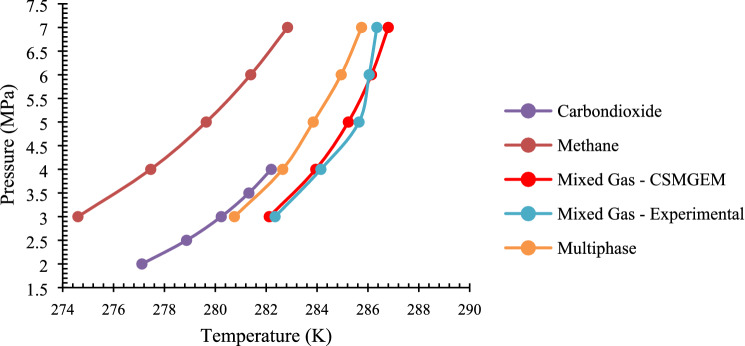
Hydrate liquid–vapor equilibrium (HLVE) curve of deionized water and crude oil systems.

Based on the experimental procedure mentioned, the experiments are conducted with a mixed gas system containing high CO_2_. All the thermodynamic points are calculated by analyzing the data logged into the system during the experimental procedure and then plotting the pressure–temperature profile. All these points are used for developing the HLVE curve. The HLVE curve to determine phase behavior is presented in Fig. 5.

To determine the effect of high CO_2_ content in the natural gas system, the comparison is made with the experimental results of methane and carbon dioxide gases. According to CSMGEM, the HLVE curve of the mixed gas system tends to move towards a higher temperature region when a fixed pressure point is considered. So, it can be concluded that the natural gas system with higher carbon dioxide tends to promote gas hydrate formation. This is because of the gas molecular structure of carbon dioxide. The size of the molecule is small compared to that of the other pure gases that are a part of general natural gas and is highly acidic. Due to this, the nucleation point of CO_2_ hydrates is also quite low compared to other gases because of which there is a brisk formation of hydrates in the presence of high CO_2_^34,35^. When a hydrate is formed in the natural gas system with high CO_2_, hydrate structure that is more viable to form is sI structure. This is due to the occupancy of CH_4_ and CO_2_ gas molecules in the larger cavities of hydrate structure. The other heavy carbon elements in composition like C_3_H_8_ and C_4_H_10,_ which are larger in molecular structure, will act as gas diluents and do not participate in the hydrate formation^36^. For the validation of the simulation, experimental data is considered and compared to that of the CSMGEM data. The results are in accordance with the simulation.

#### Multiphase system experimental analysis

Like that of the experimental procedure mentioned, the multiphase system consisting of crude oil, natural gas with high CO_2,_ and deionized water is taken. When the multiphase system is simulated with the flow conditions using magnetic stirrer with a suitable RPM, the emulsions are formed within the system. These emulsions effects the gas hydrates formation because of the Void fraction and liquid hold up^37,38^. The thermodynamic equilibrium points are determined based on the P–T curve. Then, based on these thermodynamic equilibrium points, the HLVE curve has also been developed. A comparison is made with simulation results of pure gas systems and mixed gas together with the experimental results of the mixed gas system. These results as well are included in Fig. 5 to compare it with a simple system and determine the phase behavior.

When compared with simulation results and experimental results, the HLVE curve shifted towards lower temperature and higher-pressure region. This reflects that the system is displaying an inhibition effect. This could be because of the inhibiting effect shown by more substantial inhibiting gases like nitrogen and butane reaction in the mixture. As mentioned, the nitrogen and butane tend to form gas hydrates at much higher pressures than the considered pressure range of 3–7 MPa. The interaction parameters in mixed gas that influence the solubility of gases in liquids resulting in gas hydrates formation tend to allow the lighter gases to be dissolved more when compared to that of the heavier gases^39^. This could be a reason they inhibit the formation of mixed gas hydrates with high CO_2_ content. Also, the higher stability of crude oil can be a contributing factor for hydrate inhibition. The crude oil is a viscous hydrocarbon liquid and is a raw material that contains various unprocessed salts. According to literature, the presence of polymers and salts tends to inhibit the gas hydrates formation kinetically as well as thermodynamically^40^. However, the natural gas system containing crude oil displays gas hydrate promotion behavior promote gas hydrate formation when compared to that of the pure gas systems like methane and carbon dioxide. This may occur due to the presence of a mixed gas system alongside the higher carbon chain availability in the crude oil, which results in the quicker formation of gas hydrates.

#### Temperature variance and enthalpy

The temperature variance (Ŧ) for the pure and multiphase systems are calculated to determine the effect of additional phases on the phase behavior of gas hydrates. The thermodynamic equilibrium points of the pure and multiphase gas dominant systems studied are compared and analyzed. Temperature variance (ΔT) is computed according to the following Eq. (1)

1 $$\rlap{--}T= \frac{\Delta T}{n}=\frac{\sum_{i=1}^{n}\left({T}_{0,pi}-{T}_{1,pi}\right)}{n}$$

where T_0,pi_ represents the equilibrium temperature of studied gas in the pure system, i.e., without the addition of any compound, while T_1,pi_ is the equilibrium temperature of the multiphase system. The values of both dissociation temperatures should be attained at the same pressure. The n denotes the number of pressure points taken into consideration for the study.

The results of the temperature variance are presented in Table 1.

**Table 1:** Temperature variance due to multiphase system.

Pressure (MPa)	Thermodynamic equilibrium temperature (pure system) (K)	Thermodynamic equilibrium temperature (multiphase system) (K)	Temperature variance
3	282.35	280.75	1.6
4	284.15	282.65	1.5
5	285.65	283.85	1.8
6	286.05	284.95	1.1
7	286.35	285.75	0.6
Average temperature variance			1.32

The dissociation enthalpies denoted as ΔH for gas hydrates are determined by using the Clausius–Clapeyron equation through differentiating the experimental HLVE data. The values of ΔH are achieved by calculating the slope of HLVE data attained by the Clausius–Clapeyron Eq. (2).

2 $$\frac{d\mathrm{ln}P}{d\frac{1}{T}}=\frac{\Delta H}{zR}$$

where P and T show the equilibrium pressure and temperature, respectively, R represents the universal gas constant, z is the compressibility factor of the gas involved in the study. At the same time, ΔH signifies the enthalpy of gas hydrates dissociation.

**Figure 6:**
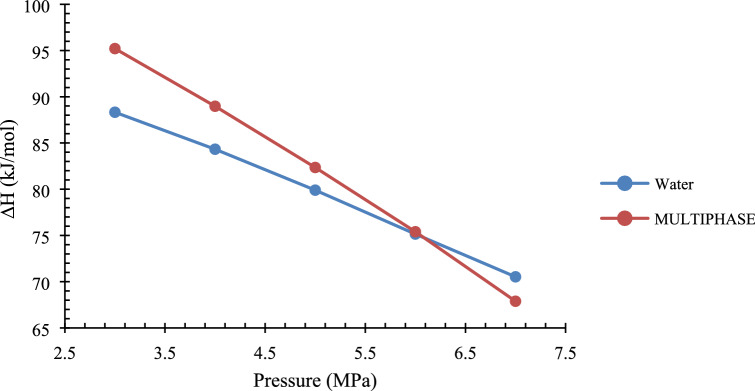
The plot of enthalpy (pure system vs. multiphase system).

The results of the enthalpy are presented in Table 2, and the graphical representation is presented in Fig. 6. It is observed that the enthalpy for the water or pure system is less than compared to that of the multiphase system. This calculation was attempted to understand further the effect of additional phases in gas hydrate formation. The presence of the oil phase in the multiphase system shows that additional energy is required to form or dissociate gas hydrates in the system. For the calculation of enthalpy, the light hydrocarbons/volatile compounds in crude oil are not considered as the carbon chain is very long and it is almost impossible to calculate compressibility factor^41–43^.

**Table 2:** Enthalpy of pure and multiphase systems.

Pressure (MPa)	Pure system	Multiphase system
3	88.33	95.21
4	84.33	88.97
5	79.90	82.35
6	75.15	75.40
7	70.53	67.89
Average (ΔH) (kJ/mol)	79.65	81.96

Based on the analysis and results from the simulation tool, it can be concluded that high CO_2_ content in the natural gas system tends to promote gas hydrate formation. This is due to the lower nucleation behavior of CO_2_ hydrates. When a hydrate is formed in the natural gas system with high CO_2_, a hydrate structure that is more viable to form is sI structure. This is due to the occupancy of CH_4_ and CO_2_ gas molecules in the larger cavities of hydrate structure. The other heavy carbon elements in composition like C_3_H_8_ and C_4_H_10,_ which are more massive in molecular structure, will act as gas diluents and do not participate in the hydrate formation^36^. Then, an additional phase of crude oil was introduced into the system. The effect of the multiphase system was studied. The results indicated that the presence of crude oil has an inhibition effect on the mixed gas HLVE phase boundary. This is because of the higher stability of crude oil that can be a contributing factor for hydrate inhibition. This is because of the presence of crude oil results in a reduction of the chemical potential of water. From the literature, crude oil has less chance of the existence of carboxylic acid groups (or if present not in a detectable amount). This was also suggested by the low acid number of crude oils. The most common functional groups observed were –OH, aromatic, and amide groups. From this and satisfactory stability of the O/W emulsion, a consensus was that the surface-active asphaltenes might be responsible for such organic inhibition^44^.

### Statistical regression analysis

#### Analysis with deionized water

Precise statistical regression analysis is also carried out for the prediction of hydrate formation temperature (HFT) at a given pressure condition theoretically. Based on the experimental results, a validation model has been developed by the optimization algorithm that helps in the prediction of HFT.

The regression analysis data of deionized water are presented in Tables 3 and 4.

**Table 3:** Regression statistics with experimental data on the pure system.

Multiple R	0.990164168
R^2^	0.980425081
Adjusted R^2^	0.973900106
Standard error	0.463137122
No. of observations	5

**Table 4:** ANOVA statistics with experimental data on the pure system.

MODEL	df	Sum of Squares	mean square	F	Significant F
Regression	1	32.2296	32.2296	150.2573	0.001169
Residual	3	0.643488	0.214496		
Total	4	32.87308			

**Figure 7:**
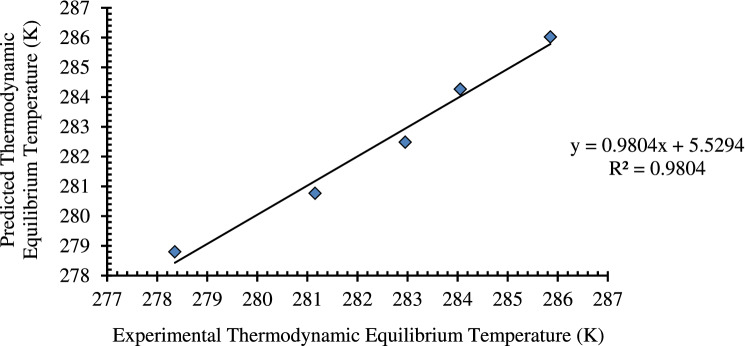
Predicted vs. actual of pure system.

The regression model predicted that the R^2^ is 0.980425081. This is also in high accordance with the adjusted R^2^ value. The standard error for the regression is predicted as 0.463137122. This implies that the regression model is significant, and the data produced by the model is accurate^45^. Based on the regression results, ANOVA statistical analysis is carried out to estimate the error between the actual vs. predicted data. The plot that suggests the actual vs. predicted is presented in Fig. 7. The plot suggests a linear regression, which means that with varying pressure, the temperature is affected or simply pressure and temperature are directly proportional to each other. Also, the equation obtained is mentioned in Fig. 7.

#### Analysis with the multiphase system

Similarly, a precise statistical regression analysis is also carried out for the prediction of hydrate formation temperature (HFT) at a given pressure condition theoretically in the multiphase system. The regression analysis data of deionized water are presented in Tables 5 and 6.

**Table 5:** Regression statistics with experimental data on the multiphase system.

Multiple R	0.980097167
R^2^	0.960590458
Adjusted R^2^	0.947453944
Standard error	0.633652262
No. of observations	5

**Table 6:** ANOVA statistics with experimental data on the multiphase system.

MODEL	df	Sum of squares	Mean Square	F	Significant F
Regression	1	29.36027	29.36027	73.1237	0.003361
Residual	3	1.204546	0.401515		
Total	4	30.56482			

**Figure 8:**
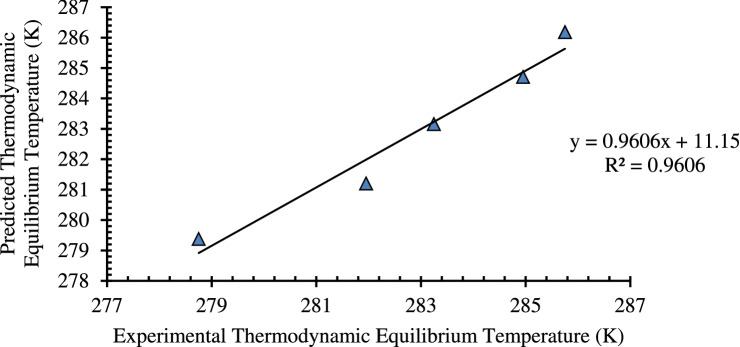
Predicted vs. actual of multiphase system.

The regression model predicted that the R^2^ is 0.960590458. This is also in high accordance with the adjusted R^2^ value. According to literature^45,46^, the model can be considered as a valid prediction model with an R^2^ value > 0.75 and adjusted R^2^ value > 0.5. Therefore, this regression analysis can be used to define HFT and HFP. The standard error for the regression is predicted as 0.633652262. This implies that the regression model is significant, and the data produced by the model is accurate. Based on the regression results, ANOVA statistical analysis is carried out to estimate the error between the actual vs. predicted data. The plot that suggests the actual vs. predicted is presented in Fig. 8. The plot suggests a linear regression, which means that with varying pressure, the temperature is affected or simply pressure and temperature are directly proportional to each other. Also, the equation obtained is mentioned in Fig. 8.

Since the gas hydrate thermodynamic experiments take approximately 48 h for each experiment, the data used for the prediction model development is limited. But the equation can be reliable and can be used as a preliminary prediction equation for the gas hydrate formation conditions prediction.

## Methodology

### Materials

The list of materials used for experimental investigation of gas hydrate formation in multiphase pipelines are as follows:

#### For equipment validation

- CH_4_
- CO_2_
- Deionized water

The gases are acquired from Gas Walker Sdn Bhd. The deionized water was prepared at gas hydrates research laboratory.

#### For phase behavior investigation

- Natural gas + deionized water
- Natural gas + deionized water + crude oil

The mixed gas system is taken in the following composition, as mentioned in Table 7.

**Table 7:** Composition of natural gas used for the study.

Gas	Composition (%)
Carbon di oxide (CO_2_)	70
Methane (CH_4_)	26
Nitrogen (N_2_)	2
Propane (C_3_H_8_)	1
Butane (C_4_H_10_)	1

The natural gas system mentioned in Table 7 is considered for experiments based on the significant components available in natural gas with high carbon dioxide (CO_2_) content. It replicates the gas composition from the K5 Gas field in Malaysia. In the K5 Gas field, natural gas is produced with high CO_2_ content. So, in this study, natural gas with high CO_2_ content has been chosen^31,47^. The Air Product Sdn. Bhd. Delivers the mixed gas system with the respective composition. The deionized water used for experimental evaluation is taken from gas hydrates research laboratory.

### Experimental apparatus

**Figure 9:**
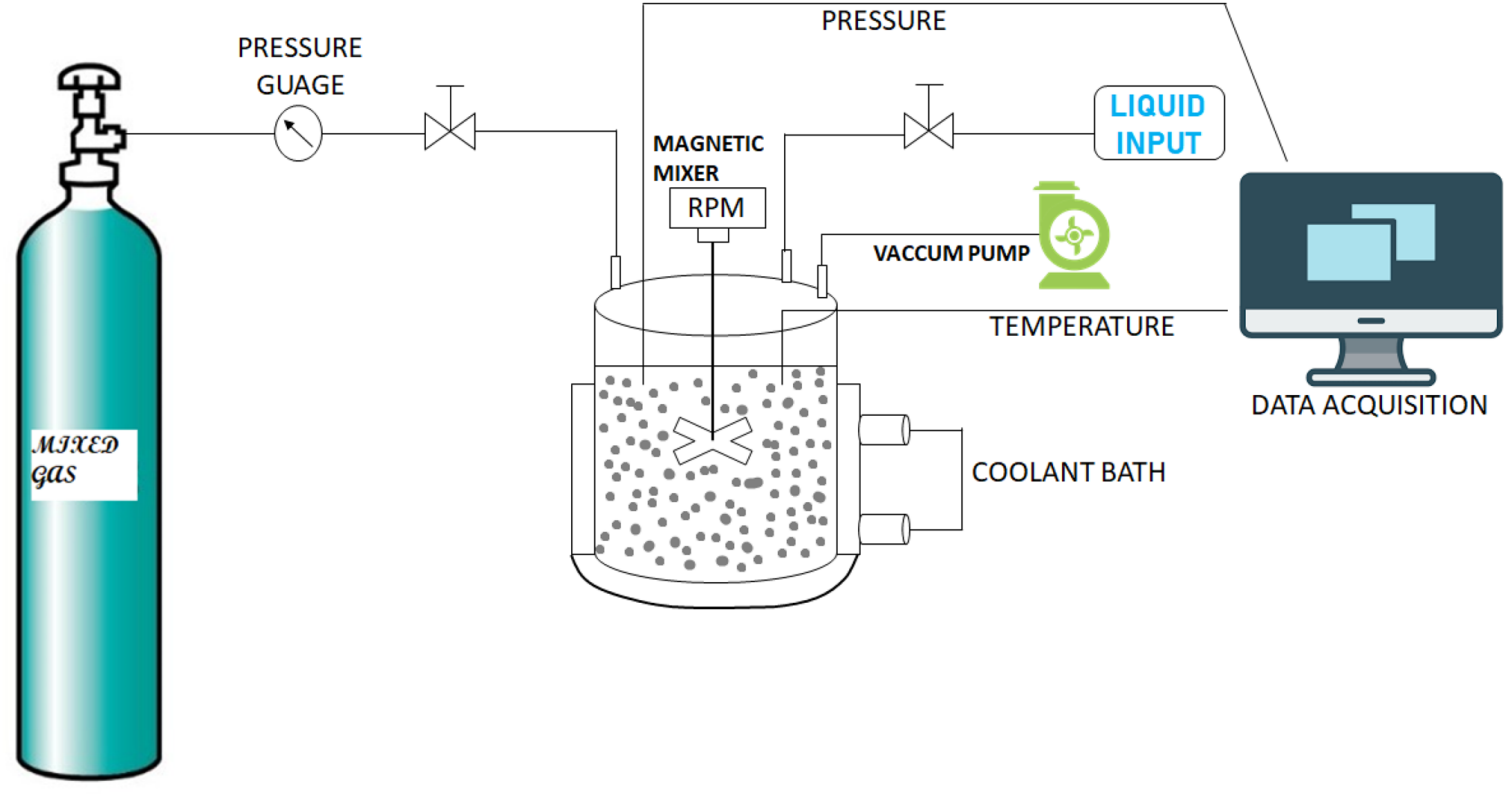
Schematic representation of the experimental setup.

Figure 9 represents the schematic representation of the experimental setup that is used in this work. The apparatus engaged for determining the phase behavior of gas hydrates in pure and multiphase systems work is equipped with a high-pressure reactor made from stainless-steel with a capacity of 650 ml. The reactor can be operated in the temperature range of – 20 to 40 °C and a pressure of 20 MPa. Pressure and temperature sensors which are connected to a data logging system are installed in the reactor to measure pressure and temperature changes for every fixed interval. The time interval for the recording was taken as 10 s. A 4-bladed impeller magnetic stirrer is installed inside the reactor to provide enough agitation during the hydrate experiment. The system temperature is controlled by a thermostatic bath equipped with a PID controller within ± 0.3 °C accuracy.

### Experimental procedure

For determining the phase equilibrium of the pure and multiphase systems, the T-cycle method with the isochoric step heating method is applied in this work. Before experiments, the reactor was washed with distilled water thoroughly to avoid any impurities and completely dried. For the pure system, a sample of 200 ml of deionized water is poured into the reactor cell. After that, the cell was inserted into the reactor and can cool down to the chosen operating condition to stabilize. Once the temperature is steadied, a small amount of gas is entered the reactor cell employing a high-pressure boosting pump. The gas that has introduced is again released to make sure that the reactor cell is vacuum. After the distinct temperature condition is achieved, gas was flowed into the reactor cell up to the anticipated pressure. In these experiments, the pressure range was chosen between 3 and 7 MPa pressures.

Once the stabilization of temperature and pressure conditions is attained, the magnetic stirrer was brought into the play by setting it at 400 RPM to disrupt the gas–liquid boundary interface. Adequate mixing is achieved during the formation of gas hydrates. According to literature, the velocity of multiphase transmission pipelines is usually maintained at 1 m/s^48–50^. To maintain this, the speed of the magnetic stirrer was chosen as 400 RPM. The quick cooling approach is utilized to lower the temperature of the reactor cell and to enhance the formation of gas hydrates. After the desired temperature is achieved, the reactor was maintained at similar conditions for an all-encompassing time (varies from 4 to 8 h). Gas hydrate formation can be confirmed with the evidence of a sharp or sudden drop in pressure with the rise in temperature in the data logging system. This is due to the exothermic reaction that takes place during gas hydrate formation. When the pressure is stabilized, and no further pressure drop is observed, it can be decided that the hydrate fully formed. Then the reactor is heated slowly with a rate of 0.5 K/h stepwise until the gas hydrate is dissociated completely. For the determination of the hydrate equilibrium point, the length of each step must be in the range of 2–6 h. The accomplishment of each experiment analysis required roughly 48 h. The same process is followed for the multiphase system as well, except the way of loading fuels into reactor cell varies. As mentioned after cleaning the cell, a sample of 100 ml of Crude oil is first poured into the cell. This crude oil was already preheated to make sure that the precipitant more massive particles get liquefied and merged with crude oil. Then deionized water of volume 200 ml is added to this.

### Statistical regression model

The experimental results are used to develop a statistical regression model to predict the gas hydrate formation conditions. Initially, the experimental results are tabulated and using data solver function, and a correlation is developed between the X and Y components. Then, the correlation equation is used to run the regression statistics. From these regression results, the prediction equation is developed to determine Y based on X. This equation is used to predict the gas hydrate formation temperature at a given pressure condition.

### CSMGEM details

For hydrate prediction, there are several software packages available for hydrate phase equilibria prediction. The commonly used hydrate prediction programs in the petroleum industry are CSMGem (CSM, USA), PVTsim (Calsep), and MultiFlash (Info-chem). However, all the commercial prediction software is only able to predict the hydrate properties for common hydrate former gases (guest molecules) in the presence of pure water and/without common hydrate inhibitors (methanol, MEG, NaCl, and KCl. The commercial prediction software is unable to accommodate (predict) the new inhibitor's behavior. The CSMGem software was developed in 2007 at the Colorado School of Mines, USA, and recognized as one of the right software among the commercial predictive soft wares. The CSMGem (the last three initials are the first letters of “Gibbs energy minimization”). The CSMGem is based upon the Gibbs energy minimization method, which allows the calculations of the formation conditions for any phase (including the hydrate). It also consents for the calculation of phases present at any temperature and pressure conditions (whether hydrates are present or not). Therefore, included are the options to perform all thermodynamic calculations with every phase and not just the hydrate.

## Conclusion

In this research work, an attempt to understand the phase behavior of gas hydrate formation in the multiphase system is made. A multiphase system with natural gas containing high CO_2_, Crude oil, and deionized water with a 20 vol% fixed water cut has been used for the experimental approach. The simulations were carried out with the CSMGEM tool, and predictions were made using the basic statistical regression model. It has been found that the presence of crude oil in the multiphase system has a suppression effect on the gas hydrate formation. Besides, it is confirmed through this work that the increment of CO_2_ level in the depleting gas well resources may lead to a surge in the occurrence of gas hydrates in the industry. The mixed gas with high CO_2_ studied displayed a promotion in gas hydrate formation in the presence of high CO_2_ content. This presents a first of its kind data with experimental, simulation, and statistical analysis on multiphase transmission systems. The difference observed in the phase behavior of the pure and multiphase system is notably significant. The authors recommend that more works are done on multiphase systems as the findings of this current work may highlight the lack of accuracy in simulation techniques of the existing software packages in predicting gas hydrate formation in multiphase flow based on the available data on single and binary phase transmission pipelines.
